# Supplementary material for: Evaluation of habitat protection under the European Natura 2000 conservation network – The example for Germany
Source: PLoS One. 2018 Dec 19;13(12):e0208264. doi: 10.1371/journal.pone.0208264 (PMC6300216; doi:10.1371/journal.pone.0208264)
Supplement: S1 File — (DOCX) [file pone.0208264.s001.docx]

S1

**Evaluation of habitat protection under the European Natura 2000 conservation network – the example for Germany**

Martin Friedrichs^1,2 *^, Virgilio Hermoso^3^, Vanessa Bremerich^1^, Simone D. Langhans^4,5,1^

^1^Department of Ecosystem Research, Leibniz-Institute of Freshwater Ecology and Inland Fisheries, Berlin, Germany

^2^ Institute of Biology, Freie Universität Berlin, Berlin, Germany

^3^ Centre Tecnològic Forestal de Catalunya (CEMFOR - CTFC), Solsona, Lleida, Spain

^4^Department of Zoology, University of Otago, Dunedin, New Zealand

^5^BC3-Basque Centre for Climate Change, Leioa, Spain

*Corresponding author:

E-mail: [friedrichs@igb-berlin.de](mailto:friedrichs@igb-berlin.de) (MF)

DETAILED INFORMATION ON MARXAN ANALYSES

*S1 Setting Marxan variables; Species penalty factor (SPF), boundary length modifier (BLM), cost, target and fixed PUs for Free-choice scenario*

Marxan solves the so called minimum set problem by trying to represent all conservation features at the minimum cost, while attending to other spatial constrains like connectivity [1].

*S1.1 Species penalty factor (SPF)*

The SPF specifies the magnitude of penalty for unmet targets [1]. In our study we set the SPF for all habitats to 10. That means the penalty for not meeting a target was the same for all habitats, and consequently, no habitat was preferred over another one in the Marxan solutions. If for example one habitat would have a higher SPF, Marxan would place a greater importance in solutions that met the target for this habitat [1]. However, a very high SPF would result in very restrictive solutions, and probably Marxan would not be able to choose PUs efficiently [1].

*S1.2 Boundary length modifier (BLM)*

As we wanted Marxan to prefer connected networks of PAs over disconnected ones, we included connectivity, via the BLM, in our analyses [1]. The higher the BLM is set, the more clumped the solutions are. The boundary length modifier for our scenarios was calibrated following the description in Stewart and Possingham [2] and was set for all scenarios to 2.

*S1.3 Defining a cost for each PU*

Human footprint values are reported on a finer scale (1 km² grid cells; Sanderson et al., 2002) than our PU layer (30 km² cells) was, therefore we used the sum of human footprint values per PU as a surrogate for cost. The result of high human footprint values in PUs is a reduced likelihood of these PUs to be chosen for the conservation network, since they are most likely heavily degraded and therefore of lower quality or very costly.

*S1.4 Defining targets for each habitat*

Targets for each habitat in Marxan were set as percent of total number of PUs in which the habitat occurs. That means a 10 % target for a habitat which occurs in 150 PUs would be set to 15 PUs. As we did not want to weight habitats differently, we included the same proportion for each habitat as a target in Marxan.

*S1.5 Fixed PUs for the free-choice scenario*

In the free-choice scenario we let Marxan freely chose from all available PUs, apart from 173 PUs located at the North and Baltic Sea, which we locked in. In these PUs that cover mainly marine habitats and only partly terrestrial ones, N2000 PAs were most likely implemented to protect habitats which we excluded from our analyses (see S1). However, due to the high coverage of marine N2000 PAs, the marine PUs were always considered as protected in all scenarios. Hence, locking these PUs in prevented against an artificially reduced performance of N2000 when compared to the free-choice scenario.

References:

1. Ball, I.R., H.P. Possingham, and M.E. Watts, *Marxan and Relatives: Software for Spatial Conservation Prioritization*. Spatial conservation prioritisation: quantitative methods and computational tools. 2009, Oxford: Oxford University Press. 185-195.

2. Stewart, R.R. and H.P. Possingham, *Efficiency, costs and trade-offs in marine reserve system design.* Environmental Modeling & Assessment, 2005. **10**(3): p. 203-213.

3. Sanderson, E.W., et al., *The human footprint and the last of the wild.* BioScience, 2002. **52**(10): p. 891-904.
