# Supplementary material for: Evaluation of habitat protection under the European Natura 2000 conservation network – The example for Germany
Source: PLoS One. 2018 Dec 19;13(12):e0208264. doi: 10.1371/journal.pone.0208264 (PMC6300216; doi:10.1371/journal.pone.0208264)
Supplement: S1 Table — Habitat classes marked with an asterisk were not used to describe Natura 2000 habitats (see explanation in text). (DOCX) [file pone.0208264.s002.docx]

S1

**Evaluation of habitat protection under the European Natura 2000 conservation network – the example for Germany**

Martin Friedrichs^1,2 *^, Virgilio Hermoso^3^, Vanessa Bremerich^1^, Simone D. Langhans^4,5,1^

^1^Department of Ecosystem Research, Leibniz-Institute of Freshwater Ecology and Inland Fisheries, Berlin, Germany

^2^ Institute of Biology, Freie Universität Berlin, Berlin, Germany

^3^ Centre Tecnològic Forestal de Catalunya (CEMFOR - CTFC), Solsona, Lleida, Spain

^4^Department of Zoology, University of Otago, Dunedin, New Zealand

^5^BC3-Basque Centre for Climate Change, Leioa, Spain

*Corresponding author:

E-mail: [friedrichs@igb-berlin.de](mailto:friedrichs@igb-berlin.de) (MF)

**S1 Table.** **EUNIS habitat classes occurring in Germany with corresponding numerical codes.** Habitat classes marked with an asterisk were not used to describe Natura 2000 habitats.

| **Code** | **Definition** |
| --- | --- |
| 11 | Coastal dunes and sandy shores |
| 14 | Surface standing waters |
| 15 | Surface running waters |
| 16 | Littoral zone of inland surface waterbodies |
| 17 | Raised and blanket bogs |
| 18 | Valley mires, poor fens and transition mires |
| 20 | Base-rich fens and calcareous spring mires |
| 21 | Sedge and reedbeds, normally without free-standing water |
| 22 | Inland saline and brackish marshes and reedbeds |
| 23 | Dry grasslands |
| 24 | Mesic grasslands |
| 25 | Seasonally wet and wet grasslands |
| 26 | Alpine and subalpine grasslands |
| 28 | Inland salt steppes |
| 31 | Arctic, alpine and subalpine scrub |
| 32 | Temperate and mediterranean-montane scrub |
| 40 | Shrub plantations |
| 41 | Broadleaved deciduous woodland |
| 43 | Coniferous woodland |
| 44 | Mixed deciduous and coniferous woodland |
| 45 | Lines of trees, small anthropogenic woodlands, recently felled woodland, early-stage woodland and coppice |
| 47 | Screes |
| 48 | Inland cliffs, rock pavements and outcrops |
| 49 | Snow or ice-dominated habitats |
| 50 | Miscellaneous inland habitats with very sparse or no vegetation |
| 52 | * Arable land and market gardens |
| 53 | * Cultivated areas of gardens and parks |
| 54 | * Buildings of cities, towns and villages |
| 55 | * Low density buildings |
| 56 | * Extractive industrial sites |
| 57 | * Transport networks and other constructed hard-surfaced areas |
| 58 | * Highly artificial man-made waters and associated structures |
| 59 | * Waste deposits |
| 101 | Littoral rock and other hard substrata |
| 102 | Littoral sediment |
| 105 | Sublittoral sediment |
| 109 | Estuaries |
| 110 | Coastal lagoons |
| 128 | Littoral rock or sediment and sea ice |
| 134 | Infralittoral and circalittoral rock and other hard substrata |
