# Supplementary material for: Evaluation of habitat protection under the European Natura 2000 conservation network – The example for Germany
Source: PLoS One. 2018 Dec 19;13(12):e0208264. doi: 10.1371/journal.pone.0208264 (PMC6300216; doi:10.1371/journal.pone.0208264)
Supplement: S2 Table — Additionally, the maximum number (n°) of PUs in which a habitat type occurs, the number of PUs covered by N2000 in each scenario (Sc. 90, Sc. 75, Sc. 50, and Sc. 25), and the number of PUs which have to be protected to reach the 17% Aichi target, are given. PU = planning unit. (DOCX) [file pone.0208264.s003.docx]

SUPPLEMENTARY INFORMATION

**Evaluation of habitat protection under the European Natura 2000 conservation network – the example for Germany**

Martin Friedrichs^1,2 *^, Virgilio Hermoso^3^, Vanessa Bremerich^1^, Simone D. Langhans^4,5,1^

^1^Department of Ecosystem Research, Leibniz-Institute of Freshwater Ecology and Inland Fisheries, Berlin, Germany

^2^ Institute of Biology, Freie Universität Berlin, Berlin, Germany

^3^ Centre Tecnològic Forestal de Catalunya (CEMFOR - CTFC), Solsona, Lleida, Spain

^4^Department of Zoology, University of Otago, Dunedin, New Zealand

^5^BC3-Basque Centre for Climate Change, Leioa, Spain

*Corresponding author:

E-mail: [friedrichs@igb-berlin.de](mailto:friedrichs@igb-berlin.de) (MF)

**S2 Table.** **Detailed description of which EUNIS habitat classes and which plant species were used to define a Natura 2000 habitat type.** Additionally, Maximum number of pus in which a habitat type occurs as well as number of pus protected in each scenario, and the number of pus which have to be protected to reach the 17% Aichi target, are given.

| Habitat code Natura 2000 | Habitat name | Eunis habitat class code | Plant species | Max. number of PUs | Number of PUs protected | | | |  |
| --- | --- | --- | --- | --- | --- | --- | --- | --- | --- |
|  |  |  |  |  | Sc. 2 | Sc. 3 | Sc. 4 | Sc. 5 | Aichi target (17%) |
| 1150 * | Coastal lagoons | 11; 14; 16; 22; 102; 110 | *Ruppia marítima, Zostera marítima, Zostera noltii, Myriophyllum spicatum, Potamogeton pectinatus, Ranunculus peltatus ssp. Baudotii, Ruppia cirrhosa, Zannichellia palustris* | 146 | 36 | 56 | 81 | 106 | 25 |
| 1210 | Annual vegetation of drift lines | 11; 101; 102; 134 | *Atriplex calotheca, Atriplex litoralis, Atriplex prostrata, Cakile maritime, Chenopodium album, Honckenya peploides, Leymus arenarius, Salsola kali, Tripleurospermum maritimum, Tripolium pannonica* | 136 | 34 | 51 | 67 | 94 | 23 |
| 1220 | Perennial vegetation of stony banks | 14; 50; 101; 102; 134 | *Atriplex litoralis, Atriplex prostrata, Cakile marítima, Crambe maritime, Elymus repens, Honckenya peploides, Leymus arenarius, Phragmites australis, Potentilla anserina, Rumex crispus* | 157 | 25 | 42 | 59 | 62 | 27 |
| 1230 | Vegetated sea cliffs of the Atlantic and Baltic Coasts | 11; 24; 28; 41; 50; 101; 134 | *Acer pseudoplatanus, Agrimonia eupatoria, Hippophae rhamnoides, Populus tremolo, Quercus robur, Sorbus aucuparia, Trifolium medium, Ulmus glabra, Festuca rubra ssp. litoralis, Tripleurospermum maritimum* | 209 | 19 | 39 | 65 | 110 | 36 |
| 1310 | Salicornia and other annuals colonizing mud and sand | 11; 16; 24; 25; 28; 102; 109; 110 | *Bolboschoenus maritimus, Glaux marítima, Salicornia europaea ssp. Brachystachya, Salicornia europaea* agg.*, Salicornia europaea ssp. Europea, Salicornia procumbens, Salicornia stricta, Spergularia media, Suaeda maritime, Tripolium pannonicum* | 208 | 73 | 105 | 147 | 173 | 35 |
| 1320 | Spartina swards (Spartinion maritimae) | 11; 16; 21; 24; 25; 28; 102; 109 | *Salicornia europaea* agg., *Salicornia europea*, *Spartina anglica*, *Sueda maritima* | 134 | 56 | 79 | 104 | 119 | 23 |
| 1330 | Atlantic salt meadows (Glauco-Puccinellietalia maritimae) | 18; 21; 22; 24; 25; 28; 109 | *Carex distans, Carex extensa, Eleocharis palustris, Eleocharis uniglumis, Elymus repens, Spergularia marina, Tripolium pannonicum* | 493 | 116 | 165 | 234 | 307 | 84 |
| 1340 * | Inland salt meadows | 22; 24; 25 | *Agrostis stolonifera*  *Festuca rubra*  *Glaux maritima*  *Juncus gerardii*  *Lotus maritimus*  *Puccinellia distans* agg.  *Puccinellia maritima*  *Salicornia europaea* agg.  *Spergularia salina*  *Tripolium pannonicum* | 343 | 10 | 17 | 43 | 80 | 58 |
| 2110 | Embryonic shifting dunes | 11; 16; 22; 23; 28; 102; 110 | *Ammophila arenaria*  *Cakile maritima*  *Carex arenaria*  *Elymus farctus ssp. borealiatlanticus*  *Eryngium maritimum*  *Festuca arundinacea*  *Festuca rubra ssp. arenaria*  *Hieracium umbellatum*  *Honckenya peploides*  *Leymus arenarius* | 146 | 56 | 75 | 92 | 111 | 25 |
| 2120 | Shifting dunes along the shoreline with Ammophila arenaria (‘white dunes’) | 11; 16; 22; 23; 28; 102; 110 | *Ammophila arenaria*  *Anthyllis vulnaria ssp. maritima*  *Carex arenaria*  *Crambe maritima*  *Eryngium maritimum*  *Festuca rubra ssp. arenaria*  *Hieracium umbellatum*  *Honckenya peploides*  *Leymus arenarius*  *Petasites spurius* | 163 | 57 | 75 | 100 | 128 | 28 |
| 2130 * | Fixed coastal dunes with herbaceous vegetation (‘grey dunes’) | 11; 21; 22; 23; 24; 28; 110 | *Aira caryophyllea*  *Aira praecox*  *Carex arenaria*  *Cerastium diffusum*  *Corynephrus canescens*  *Festuca rubra ssp. arenaria*  *Galium verum*  *Ornithopus perpusillus*  *Silene otitis*  *Thymus serpyllum* | 174 | 55 | 77 | 107 | 134 | 30 |
| 2140 * | Decalcified fixed dunes with Empetrum nigrum | 11; 23; 24; 28; 32 | *Deschampsia flexuosa*  *Empetrum nigrum*  *Hieracium pilosella*  *Hieracium umbellatum*  *Hypochaeris radicta*  *Polypodium vulgare*  *Potentilla erecta*  *Salix repens*  *Vaccinium myrtillus*  *Veronica officinalis* | 66 | 36 | 47 | 57 | 65 | 11 |
| 2150 * | Atlantic decalcified fixed dunes (Calluno-Ulicetea) | 11; 23; 24; 28; 32 | *Calluna vulgaris*  *Carex arenaria*  *Empetrum nigrum* agg.  *Erica tetralix*  *Hieracium umbellatum*  *Juncus squarrosus*  *Molina caerulea* agg.  *Potentilla erecta*  *Rumex acetosella*  *Salix repens* | 74 | 21 | 34 | 47 | 58 | 13 |
| 2160 | Dunes with Hippophaë rhamnoides | 11; 23; 24; 28; 32 | *Carex arenaria*  *Crataegus monogyna*  *Epilobium angustifolium*  *Hippophae rhamnoides*  *Prunus spinosa* agg.  *Rosa canina*  *Rubus caesius*  *Salix repens*  *Sambucus nigra*  *Solanum dulcamara* | 95 | 32 | 48 | 62 | 71 | 16 |
| 2170 | Dunes with Salix repens ssp. argentea (Salicion arenariae) | 11; 24; 28; 32 | *Carex arenaria*  *Epipactis helleborine*  *Polypodium vulgare*  *Pyrola minor*  *Pyrola rotundifolia*  *Rosa pimpinellifolia*  *Rubus caesius*  *Salix repens*  *Thalictrum minus* | 75 | 36 | 48 | 57 | 63 | 13 |
| 2180 | Wooded dunes of the Atlantic, Continental and Boreal region | 11; 24; 25; 41; 44 | *Alnus glutinosa*  *Betula pendula*  *Betula pubescens ssp. pubescens*  *Calamagrostis cenescens*  *Empetrum nigrum*  *Erica tetralix*  *Pinus sylvestris*  *Polypodium vulgaris*  *Quercus robur*  *Sorbus aucuparia* | 114 | 35 | 52 | 68 | 86 | 19 |
| 2190 | Humid dune slacks | 11; 21; 24; 25; 32; 41 | *Centaurium littorale*  *Epilobium hirsutum*  *Juncus balticus*  *Littorea uniflora*  *Myrica gale*  *Potentilla erecta*  *Pyrola rotundiflora*  *Sagina nodosa*  *Salix repens*  *Typha latifolia* | 99 | 40 | 55 | 66 | 80 | 17 |
| 2310 | Dry sand heaths with Calluna and Genista | 23; 24; 32 | *Arctostaphylos urva-ursi*  *Calluna vulgaris*  *Cuscuta epithymum*  *Empetrum nigrum* agg.  *Genista anglica*  *Genista germanica*  *Genista pilosa*  *Lycopodium clavatum*  *Vaccinium myrtillus*  *Vaccinium vitis-idaea* | 733 | 39 | 64 | 124 | 236 | 125 |
| 2320 | Dry sand heaths with Calluna and Empetrum nigrum | 24; 32 | *Calluna vulgaris*  *Carex arenaria*  *Cuscuta epithymum*  *Empetrum nigrum*  *Empetrum nigrum* agg.  *Erica tetralix*  *Genista anglica*  *Genista pilosa*  *Vaccinium myrtillus*  *Vaccinium vitis-idaea* | 85 | 2 | 2 | 4 | 11 | 14 |
| 2330 | Inland dunes withopen Corynephorus and Agrostis grasslands | 23; 24; 50 | *Aira caryophyllea*  *Aira praecox*  *Armeria halleri*  *Artemisia campestris*  *Corynephrus canescens*  *Dianthus deltoids*  *Filago minima*  *Rumex acetosella*  *Spergula mosisonii*  *Teesdalia nudicaulis* | 1194 | 59 | 99 | 197 | 374 | 203 |
| 3110 | Oligotrophic waters containing very few minerals of sandy plains (Littorelletalia uniflorae) | 14 | *Baldellia ranunculoides*  *Deschampsia setacea*  *Eleocharis multicaulis*  *Isoetes echinospora*  *Isoetes lacustris*  *Juncus bulbosus*  *Lobelia dortmanna*  *Myriophyllum alterniflorum*  *Pilularia globulifera*  *Ranunculus ololeucos* | 16 | 0 | 1 | 1 | 4 | 3 |
| 3130 | Oligotrophic to mesotrophic standing waters with vegetation of the Littorelletea uniflorae and/or of the Isoëto-Nanojuncetea | 14; 16; 18; 20; 21; 25 | *Centaurium pulchellum*  *Cyperus fuscus*  *Eleocharis multicaulis*  *Gnaphalium uliginosum*  *Heloscadium inundatum*  *Hypericum humifusum*  *Isolepsis setacea*  *Juncus bulbosus*  *Littorea uniflora*  *Potamogeton polygonifolius* | 700 | 32 | 60 | 123 | 255 | 119 |
| 3140 | Hard oligo-mesotrophic waters with benthic vegetation of Chara spp. | 14 | *Hippuris vulgaris*  *Nuphar lutea*  *Potamogeton alpinus*  *Potamogeton coloratus*  *Potamogeton friesii*  *Potamogeton gramineus*  *Potamogeton lucens*  *Potamogeton natans*  *Potamogeton trichoides*  *Ranunculus trichophyllus* | 698 | 48 | 85 | 183 | 334 | 119 |
| 3150 | Natural eutrophic lakes with Magnopotamion or Hydrocharition - type vegetation | 14; 15; 16 | *Azolla filiculoides*  *Callitriche stagnalis*  *Ceratophyllum demersum*  *Hippuris vulgaris*  *Hydrocharis morsus- ranae*  *Lemna minor*  *Spirodela polyrhiza*  *Stratiotes aloides*  *Utricularia australis*  *Utricularia vulgaris* | 2046 | 74 | 141 | 333 | 712 | 348 |
| 3160 | Natural dystrophic lakes and ponds | 14; 17; 18; 20 | *Carex lasiocarpa*  *Carex rostrata*  *Drosera intermedia*  *Eleocharis multicaulis*  *Eriophorum angustifolium*  *Juncus bulbosus*  *Nymphaea candida*  *Rhynchospora fusca*  *Scheuchzeria palustris*  *Utricularia vulgaris* | 746 | 41 | 77 | 141 | 266 | 127 |
| 3180 * | Turloughs | 14 |  | 11 | 0 | 0 | 0 | 6 | 2 |
| 3190 | Lakes of gypsum karst | 14 |  | 8 | 0 | 0 | 0 | 3 | 1 |
| 3220 | Alpine rivers and the herbaceous vegetation along their banks | 14; 15; 50 | *Buphthalmum salicifolium*  *Campanula cochleariifolia*  *Chondrilla chondrilloides*  *Dryas octopetalia*  *Epilobium fleischeri*  *Gypsophila repens*  *Linaria alpina*  *Saxifraga aizoides*  *Saxifraga caesia*  *Thymus praecox* | 58 | 5 | 11 | 19 | 30 | 10 |
| 3230 | Alpine rivers and their ligneous vegetation with Myricaria germanica | 14; 15; 50 | *Buphthalmum salicifolium*  *Calamogrostis phragmitoides*  *Campanula cochleariifolia*  *Chondrilla chondrilloides*  *Epilobium fleischeri*  *Gypsophila repens*  *Linaria alpina*  *Salix daphnoides*  *Salix eleagnos*  *Salix purpurea* | 25 | 1 | 4 | 8 | 11 | 4 |
| 3240 | Alpine rivers and their ligneous vegetation with Salix elaeagnos | 14; 15; 31; 32; 50 | *Agrostis gigantea*  *Alnus incana*  *Campanula cochleariifolia*  *Equisetum variegatum*  *Hippophae rhamnoides*  *Linaria alpina*  *Salix daphnoides*  *Salix eleagnos*  *Salix purpurea*  *Thymus praecox* | 113 | 10 | 18 | 33 | 55 | 19 |
| 3260 | Water courses of plain to montane levels with the Ranunculion fluitantis and Callitricho-Batrachion vegetation | 14; 15 | *Berula erecta*  *Callitriche hamulata*  *Potamogeton berchtoldii*  *Potamogeton crispus*  *Potamogeton pectinatus*  *Potamogeton nodosus*  *Ranunculus fluitans*  *Ranunculus peltatus*  *Sparganium emersum*  *Veronica beccabunga* | 1872 | 67 | 123 | 290 | 629 | 318 |
| 3270 | Rivers with muddy banks with Chenopodion rubri p.p. and Bidention p.p. vegetation | 14; 15; 16; 21; 50 | *Bidens frondosa*  *Brassica nigra*  *Chenopodium rubrum*  *Chenopodium ficifolium*  *Chenopodium glaucum*  *Corigiola littoralis*  *Ranunculus sceleratus*  *Rorippa palustris*  *Xanthium albinum* | 445 | 6 | 16 | 62 | 180 | 76 |
| 4010 | Northern Atlantic wet heaths with Erica tetralix | 17; 18; 24; 25 | *Drosera rotundifolia*  *Erica tetralix*  *Eriophorum angustifolium*  *Eriophorum vaginatum*  *Juncus squarrosus*  *Molinia caerulea*  *Narthecium ossifragum*  *Trichophorum germanicum*  *Vaccinium uliginosum* | 707 | 22 | 41 | 72 | 149 | 120 |
| 4030 | European dry heaths | 23; 24; 32; 40 | *Calluna vulgaris*  *Cuscuta epithymum*  *Festuca ovina*  *Genista anglica*  *Genista germanica*  *Genista pilosa*  *Lycopodium clavatum*  *Rumex acetosella*  *Vaccinium myrtillus*  *Vaccinium vitis-idaea* | 4100 | 87 | 193 | 446 | 974 | 697 |
| 4060 | Alpine and Boreal heaths | 26; 31 | *Arctostaphylos uva- ursi*  *Dryas octopetalia*  *Empetrum hermaphroditum*  *Erica carnea*  *Lonicera caerulea*  *Nardus stricta*  *Rhododendron ferrugineum*  *Rhododendron hirsutum*  *Vaccinium myrtillus*  *Vaccinium vitis-idaea* | 105 | 9 | 17 | 36 | 53 | 18 |
| 4070 * | Bushes with Pinus mugo and Rhododendron hirsutum (Mugo-Rhododendretum hirsuti) | 31; 32; 43 | *Calamagrostis varia*  *Erica carnea*  *Huperzia selago*  *Lyopodium annotinum*  *Pinus mugo*  *Polygala chamaebuxus*  *Rhododendron ferrugineum*  *Rhododendron hirsutum*  *Vaccinium myrtillus*  *Vaccinium vitis-idaea* | 158 | 11 | 21 | 44 | 71 | 27 |
| 4080 | Sub-Arctic Salix spp. scrub | 31; 32; 41 | *Salix glabra*  *Salix hastata*  *Salix waldsteiniana*  *Sorbus chamaemespilus* | 54 | 5 | 9 | 19 | 28 | 9 |
| 40A0 * | Subcontinental peri-Pannonic scrub | 32; 40; 41 | *Amelanchier embergeri*  *Cotoneaster integerrimus*  *Ligustrum vulgare*  *Prunus fruticosa*  *Prunus mahaleb*  *Rhamnus cathartica*  *Rosa rubiginosa*  *Rosa spinosissima*  *Sorbus torminalis*  *Viburnum lantana* | 144 | 0 | 1 | 10 | 41 | 24 |
| 5110 | Stable xerothermophilous formations with Buxus sempervirens on rock slopes (Berberidion p.p.) | 40; 41 | *Amelanchier embergeri*  *Berberis vulgaris*  *Buxus sempervirens*  *Clematis vitalba*  *Corylus avellana*  *Dictamnus albus*  *Geranium sanguineum*  *Ligustrum vulgare*  *Peucedanum cervaria*  *Prunus mahaleb* | 20 | 0 | 0 | 3 | 11 | 3 |
| 5130 | Juniperus communis formations on heaths or calcareous grasslands | 23; 24; 26; 31; 32; 40 | *Calluna vulgaris*  *Carex caryophyllea*  *Carlina acaulis*  *Helictotrichon pratense*  *Hypericum maculatum*  *Juniperus communis*  *Koeleria macrantha*  *Orchis militaris*  *Vaccinium myrtillus*  *Viola canina* | 1524 | 32 | 84 | 193 | 408 | 259 |
| 6110 * | Rupicolous calcareous or basophilic grasslands of the Alysso-Sedion albi | 24; 50 | *Alyssum alyssoides*  *Alyssum montanum*  *Cerastium brachypetalum*  *Cerastium pumilum*  *Erysimum crepidifolium*  *Festuca pallens*  *Melica ciliata*  *Saxifraga tridactylites*  *Sedum acre*  *Thlaspi perfoliatum* | 1276 | 11 | 35 | 106 | 286 | 217 |
| 6120 * | Xeric sand calcareous grasslands | 23; 24; 50 | *Armeria maritima*  *Centaurea stoebe*  *Dianthus deltoides*  *Festuca ovina*  *Festuca psammophila*  *Koeleria glauca*  *Koeleria macrantha*  *Medicago minima*  *Onosma arenaria*  *Silene otites* | 1066 | 77 | 131 | 251 | 438 | 181 |
| 6130 | Calaminarian grasslands of the Violetalia calaminariae | 24; 25; 56 | *Armeria maritima*  *Armeria maritima ssp. elongata*  *Cardaminopsis halleri*  *Festuca brevipila*  *Festuca guestfalica*  *Festuca ovina*  *Minurta caespitosa*  *Silene vulgaris*  *Viola calaminaria*  *Viola guestphalica* | 181 | 0 | 1 | 10 | 30 | 31 |
| 6150 | Siliceous alpine and boreal grasslands | 24; 25; 26 | *Agrostis rupestris*  *Campanula barbata*  *Diphasiastrum alpinum*  *Gentiana acaulis*  *Gentiana punctata*  *Hypochaeris uniflora*  *Leontodon helvetica*  *Phyteuma betonicifolium*  *Pulsatilla apiifolia*  *Vernoica bellidioides* | 96 | 10 | 18 | 39 | 56 | 16 |
| 6170 | Alpine and subalpine calcareous grasslands | 24; 25; 26 | *Arabis caerulea*  *Carex ferruginea*  *Carex sempervirens*  *Dryas octopetalia*  *Festuca quadriflora*  *Kobresia myosuroides*  *Leontopodium alpinum*  *Pedicularis foliosa*  *Phleum hirsutum*  *Saxifraga androsacea* | 152 | 10 | 19 | 42 | 70 | 26 |
| 6210 | Semi-natural dry grasslands and scrubland facies on calcareous substrates (Festuco-Brometalia) (* important orchid sites) | 23; 24; 26 | *Anacampestris pyramidalis*  *Anthyllis vulneraria*  *Biscutella laevigata*  *Bromus erectus*  *Koeleria pyramidata*  *Medicago falcata*  *Orchis militaris*  *Oxytropis pilosa*  *Stipa capillata*  *Stipa pulcherrima* | 3959 | 70 | 162 | 424 | 965 | 673 |
| 6230 * | Species-rich Nardus grasslands, on silicious substrates in mountain areas (and submountain areas in Continental Europe) | 23; 24; 26 | *Arnica montana*  *Galium saxatile*  *Hypericum maculatum*  *Juncus squarrosus*  *Nardus stricta*  *Pedicularis sylvatica*  *Polygala serpyllifolia*  *Polygala vulgaris*  *Pseudorchis albida*  *Viola canina* | 3156 | 68 | 156 | 385 | 800 | 537 |
| 6240 * | Sub-Pannonic steppic grasslands | 23; 24 | *Adonis vernalis*  *Agrimonia eupatoria*  *Allium sphaerocephalon*  *Anthericum ramosum*  *Anthyllis vulneraria*  *Fragaria viridis*  *Onobrychis viciifolia*  *Seseli hippomarathrum*  *Stipa capillata*  *Thesium linophyllon* | 492 | 19 | 33 | 82 | 162 | 84 |
| 6410 | Molinia meadows on calcareous, peaty or clayey-silt-laden soils (Molinion caeruleae) | 24; 25; 26 | *Allium angulosum*  *Betonica officinalis*  *Caltha palustris*  *Carex nigra*  *Cirsium tuberosum*  *Geum rivale*  *Iris sibirica*  *Juncus acutiflorus*  *Silaum silaus*  *Valeriana pratensis* | 3692 | 110 | 216 | 502 | 1062 | 628 |
| 6430 | Hydrophilous tall herb fringe communities of plains and of the montane to alpine levels | 16; 21; 24; 25 | *Adenostyles alliariae*  *Angelica archangelica*  *Calamagrostis villosa*  *Chaerophyllum bulbosum*  *Chelidonium majus*  *Epilobium hirsutum*  *Filipendula ulmaria*  *Lamium maculatum*  *Sonchus palustris* | 8630 | 138 | 319 | 820 | 1842 | 1467 |
| 6440 | Alluvial meadows of river valleys of the Cnidion dubii | 21; 24; 25 | *Carex cespitosa*  *Deschampsia cespitosa*  *Gratiola officinalis*  *Iris pseudacorus*  *Lathyrus palustris*  *Sanguisorba officinalis*  *Scutellaria hastifolia*  *Silam silaus*  *Viola pumila*  *Viola persicifolia* | 525 | 28 | 49 | 108 | 227 | 89 |
| 6510 | Lowland hay meadows (Alopecurus pratensis, Sanguisorba officinalis) | 23; 24; 25; 26 | *Alopecurus pratensis*  *Arrhenatherum elatius*  *Crepsis biennis*  *Festuca rubra*  *Gallium mollugo*  *Geranium pratense*  *Poa pratensis*  *Polygala vulgaris*  *Ranunculus repens*  *Trisetum flavescens* | 7851 | 140 | 319 | 801 | 1779 | 1335 |
| 6520 | Mountain hay meadows | 24; 26 | *Astrantia major*  *Centaurea nigra*  *Centaurea pseudophrygia*  *Crepsis mollis*  *Geranium sylvaticum*  *Lathyrus linifolius*  *Meum athamanticum*  *Poa chaixii*  *Trifolium montanum*  *Trisetum flavescens* | 1208 | 27 | 74 | 185 | 366 | 205 |
| 7110 * | Active raised bogs | 14; 17; 18; 20; 21; 45 | *Andromeda polifolia*  *Betula nana*  *Drosera intermedia*  *Drosera rotundifolia*  *Rhynchospora alba*  *Rubus chamaemorus*  *Scheuchzeria palustris*  *Utricularia minor*  *Utricularia stygia*  *Vaccinium oxycoccos* | 329 | 4 | 14 | 44 | 112 | 56 |
| 7120 | Degraded raised bogs still capable of natural regeneration | 17; 18; 20; 25; 45 | *Agrostis canina*  *Betula nana*  *Carex echinata*  *Eriophorum angustifolium*  *Rhynchospora alba*  *Rubus chamaemorus*  *Scheuchzeria palustris*  *Utricularia minor*  *Utricularia stygia*  *Vaccinium oxycoccos* | 800 | 17 | 29 | 79 | 187 | 136 |
| 7140 | Transition mires and quaking bogs | 14; 16; 17; 18; 20; 21; 25 | *Andromeda polifolia*  *Carex lasiocarpa*  *Carex rostrata*  *Eriophorum gracile*  *Lysimachia thyrsiflora*  *Peucedanum palustre*  *Scheuchzeria palustris*  *Utricularia minor*  *Vaccinium oxycoccos* | 1993 | 85 | 156 | 328 | 651 | 339 |
| 7150 | Depressions on peat substrates of the Rhynchosporion | 17; 18; 20; 25 | *Carex limosa*  *Drosera anglica*  *Drosera intermedia*  *Drosera rotundifolia*  *Juncus bulbosus*  *Lycopodiella inundata*  *Potentilla palustris*  *Rhynchospora alba*  *Rhynchospora fusca*  *Scheuchzeria palustris* | 584 | 17 | 32 | 64 | 152 | 99 |
| 7210 * | Calcareous fens with Cladium mariscus and species of the Caricion davallianae | 16; 17; 18; 20; 21; 25 | *Carex davalliana*  *Carex nigra*  *Cladium mariscus*  *Epipactis palustris*  *Eriophorum latifolium*  *Hydrocotyle vulgaris*  *Mentha aquatica*  *Phragmites australis*  *Scheuchzeria palustris*  *Viola palustris* | 409 | 33 | 58 | 112 | 188 | 70 |
| 7220 * | Petrifying springs with tufa formation (Cratoneurion) | 14; 15; 20; 25 | *Cardamine amara*  *Chrysosplenium alternifolium*  *Cochleria pyrenaica*  *Epilobium alsinifolium*  *Nasturtium officinale* agg.  *Pinguicula vulgaris*  *Saxifrag aizoides*  *Saxifraga stellaris*  *Stellaria alsine*  *Veronica beccabunga* | 736 | 12 | 31 | 77 | 191 | 125 |
| 7230 | Alkaline fens | 17; 18; 20; 21, 25 | *Carex davalliana*  *Carex lepidocarpa*  *Carex nigra*  *Carex pulicaris*  *Dactylorhiza incarnata*  *Eleocharis quinqueflora*  *Juncus subnodulosus*  *Parnassia palustris*  *Schoenus ferrugineus*  *Schoenus nigricans* | 903 | 31 | 67 | 148 | 294 | 154 |
| 7240 * | Alpine pioneer formations of the Caricion bicoloris-atrofuscae | 24; 26 | *Carex capillaris*  *Carex firma*  *Carex frigida*  *Carex nigra*  *Equisetum variegatum*  *Eriophorum angustifolium*  *Juncus articulatus*  *Juncus triglumis*  *Kobresia simpliciuscula*  *Tofieldia pusilla* | 50 | 7 | 12 | 23 | 32 | 9 |
| 8110 | Siliceous scree of the montane to snow levels (Androsacetalia alpinae and Galeopsietalia ladani) | 24; 26; 48; 50 | *Asplenium septentrionale*  *Cerastium uniflorum*  *Cryptogamma crispa*  *Geum reptans*  *Luzula alpinopilosa*  *Oxyria digyna*  *Ranunculus glacialis*  *Saxifraga bryoides*  *Sedum alpestre*  *Sedum rupestre* | 36 | 1 | 6 | 10 | 18 | 6 |
| 8120 | Calcareous and calcshist screes of the montane to alpine levels (Thlaspietea rotundifolii) | 24; 26, 47; 48; 50 | *Achillea atrata*  *Cystopteris montana*  *Dryopteris villarii*  *Gymnocarpium robertianum*  *Gypsophila repens*  *Leontodon hispidus ssp. hyoseroides*  *Noccaea rotundifolia ssp. rotundifolia*  *Petasites paradoxus*  *Sesleria ovata*  *Valeriana montana* | 159 | 11 | 20 | 44 | 72 | 27 |
| 8150 | Medio-European upland siliceous screes | 24; 48; 50 | *Anarrhinum bellidifolium*  *Epilobium collinum*  *Epilobium lanceolatum*  *Galeopsis angustifolia*  *Galeopsis ladanum*  *Galeopsis segetum*  *Gymnocarpium robertianum*  *Rumex scutatus*  *Senecio viscosus*  *Teucrium botrys* | 1258 | 17 | 55 | 155 | 353 | 214 |
| 8160 * | Medio-European calcareous scree of hill and montane levels | 23; 24; 47; 48; 50 | *Aethionema saxatile*  *Cardaminopsis arenosa*  *Cystopteris fragilis* agg.  *Galeopsis angustifolia*  *Galeopsis ladanum*  *Gymnocarpium robertianum*  *Petasites paradoxus*  *Rumex scutatus*  *Stipa calamagrostis*  *Vincetoxicum hirudinea* | 1310 | 18 | 50 | 147 | 349 | 223 |
| 8210 | Calcareous rocky slopes with chasmophytic vegetation | 23; 24; 26; 47; 48, 50 | *Asplenium ceterach*  *Asplenium ruta- muraria*  *Asplenium trichomanes*  *Cardaminopsis patraea*  *Kernera saxitilis*  *Rhamnus pumila*  *Saxifraga paniculata*  *Saxifraga rosacea*  *Sedum dasyphyllum*  *Valeriana tripteris* | 1905 | 25 | 68 | 184 | 451 | 324 |
| 8220 | Siliceous rocky slopes with chasmophytic vegetation | 24; 48, 50 | *Asplenium adiantum- nigrum*  *Asplenium adulterinum*  *Asplenium cuneifolium*  *Asplenium septentrionale*  *Asplenium trichomanes*  *Asplenium viride*  *Epilobium collinum*  *Polypodium vulgare*  *Sedum rupestre*  *Woodsia ilvensis* | 1989 | 18 | 63 | 180 | 461 | 338 |
| 8230 | Siliceous rock with pioneer vegetation of the Sedo-Scleranthion or of the Sedo albi-Veronicion dillenii | 24; 50 | *Cerastium glutinosum*  *Festuca pallens*  *Gagea saxitalis*  *Melica ciliata*  *Myosotis ramosissima*  *Sedum album*  *Sedum annuum*  *Sempervirum arachnoideum*  *Veronica dillenii*  *Veronica verna* | 969 | 6 | 24 | 88 | 227 | 165 |
| 8340 | Permanent glaciers | 49; 50 | *Arabis caerulea*  *Campanula cochleariifolia*  *Carex parviflora*  *Leontodon montana ssp. melanotricha*  *Linaria alpina*  *Minuartia sedoides*  *Moehringia ciliata*  *Saxifraga androsacea*  *Saxifraga moschata* | 6 | 2 | 3 | 3 | 4 | 1 |
| 9110 | Luzulo-Fagetum beech forests | 41; 44 | *Abies alba*  *Acer pseudoplatanus*  *Betula pubescens ssp. pubescens*  *Calamagrostis villosa*  *Fagus sylvatica*  *Picea abies*  *Quercus petraea*  *Quercus robur*  *Sorbus aucuparia*  *Vaccinium myrtillus* | 7850 | 164 | 336 | 801 | 1714 | 1335 |
| 9120 | Atlantic acidophilous beech forests with Ilex and sometimes also Taxus in the shrublayer (Quercion robori-petraeae or Ilici-Fagenion) | 41; 44 | *Betula pendula*  *Carex pilulifera*  *Hypericum pulchrum*  *Ilex aquifolium*  *Populus tremola*  *Quercus petraea*  *Quercus robur*  *Sorbus aucuparia*  *Taxus baccata*  *Veronica officinalis* | 238 | 0 | 2 | 6 | 16 | 40 |
| 9130 | Asperulo-Fagetum beech forests | 41; 44 | *Abies alba*  *Acer pseudoplatanus*  *Betula pendula*  *Carpinus betulus*  *Fagus sylvatica*  *Fraxinius excelsior*  *Populus tremola*  *Prunus avium*  *Taxus baccata*  *Tilia cordata* | 6786 | 138 | 294 | 733 | 1592 | 1154 |
| 9140 | Medio-European subalpine beechwoods with Acer and Rumex arifolius | 41; 44 | *Acer pseudoplatanus*  *Adenostyles alliariae*  *Chaerophyllum villarsii*  *Cicerbita alpina*  *Fagus sylvatica*  *Lilium martagon*  *Polygonatum verticillatum*  *Ranunculus platanifolius*  *Rosa pendulina*  *Senecio nemorensis* agg. | 169 | 10 | 17 | 41 | 80 | 29 |
| 9150 | Medio-European limestone beechforests of the Cephalanthero-Fagion | 41; 44 | *Campanula persicifolia*  *Carex alba*  *Carex humilis*  *Carex montana*  *Cephalanthera damasonium*  *Cephalanthera rubra*  *Fagus sylvatica*  *Primula veris*  *Sesleria albicans*  *Sorbus torminalis* | 1722 | 24 | 69 | 201 | 443 | 293 |
| 9160 | Sub-Atlantic oak-hornbeam forests (Stellario-Carpinetum) | 41 | *Carpinus betulus*  *Convallaria majalis*  *Fraxinius excelsior*  *Potentilla sterilis*  *Primula elatior*  *Quercus robur*  *Ranunculus auricomus* agg.  *Stellaria holostea*  *Stellaria nemorum*  *Tilia cordata* | 5710 | 127 | 262 | 610 | 1324 | 971 |
| 9170 | Galio-Carpinetum oak-hornbeam forests | 41 | *Asarum eurpaeum*  *Campanula persicifolia*  *Carpinus betulus*  *Convallaria majalis*  *Galium sylvaticum*  *Quercus petraea*  *Sorbus domestica*  *Sorbus torminalis*  *Viburnum lantana*  *Vinca minor* | 3467 | 61 | 119 | 350 | 827 | 589 |
| 9180 * | Tilio-Acerion forests of slopes, screes and ravines | 41; 44 | *Acer platanoides*  *Actea spicata*  *Asplenium scolopendrium*  *Campanula latifolia*  *Centaurea montana*  *Polystichum aculeatum*  *Tilia cordata*  *Tilia platyphyllos*  *Ulmus glabra*  *Viola mirabilis* | 3912 | 81 | 174 | 444 | 980 | 665 |
| 9190 | Old acidophilous oak woods with Quercus robur on sandy plains | 41; 44 | *Agrostis capillaris*  *Betula pendula*  *Calamagrostis arundinacea*  *Carex pilulifera*  *Luzula luzuloides*  *Pinus sylvestris*  *Quercus petraea*  *Quercus robur*  *Vaccinium vitis- idaea*  *Veronica officinalis* | 3152 | 122 | 214 | 431 | 835 | 536 |
| 91D0 * | Bog woodland | 41; 43; 44 | *Agrostis canina*  *Andromeda polifolia*  *Betula pubescens ssp. pubescens*  *Erica tetralix*  *Eriophorum vaginatum*  *Myrica gale*  *Pinus cembra*  *Pinus mugo* agg.  *Pinus sylvestris*  *Vaccinium uliginosum* | 3521 | 136 | 252 | 522 | 1022 | 599 |
| 91E0 * | Alluvial forests with Alnus glutinosa and Fraxinus excelsior (Alno-Padion, Alnion incanae, Salicion albae) | 41 | *Alnus incana*  *Carex remota*  *Circaea lutetiana*  *Prunus padus*  *Ribes rubrum*  *Salix alba*  *Salix fragilis*  *Salix pentandra*  *Salix purpurea*  *Stellaria nemorum* | 8830 | 151 | 335 | 846 | 1926 | 1501 |
| 91F0 | Riparian mixed forests of Quercus robur, Ulmus laevis and Ulmus minor, Fraxinus excelsior or Fraxinus angustifolia, along the great rivers (Ulmenion minoris) | 41 | *Carpinus betulus*  *Clematis vitalba*  *Filipendula ulmaria*  *Fraxinus excelsior*  *Phalaris arundinacea*  *Prunus avium*  *Quercus robur*  *Stellaria holostea*  *Ulmus laevis*  *Ulmus minor* | 1116 | 22 | 40 | 109 | 295 | 190 |
| 91G0 * | Pannonic woods with Quercus petraea and Carpinus betulus | 41 | *Campanula persicifolia*  *Carpinus betulus*  *Corydalis intermedia*  *Galium intermedium*  *Hepatica nobilis*  *Polygonatum odoratum*  *Quercus petraea*  *Quercus robur*  *Rhamnus cathartica*  *Tilia cordata* | 34 | 2 | 2 | 4 | 11 | 6 |
| 91T0 | Central European lichen Scots pine forests | 43; 44 | *Agrostis capillaris*  *Calluna vulgaris*  *Carex pilulifera*  *Corynephrus canescens*  *Deschampsia flexuosa*  *Erica carnea*  *Festuca ovina*  *Hieracium pilosella*  *Pinus sylvestris*  *Vaccinium myrtillus* | 301 | 12 | 28 | 56 | 105 | 51 |
| 91U0 | Sarmatic steppe pine forest | 43 | *Coronilla vaginalis*  *Dianthus arenarius*  *Gysophila festigiata*  *Helichrysum arenarium*  *Molina caerulea*  *Peucedanum oreoselinum*  *Pinus sylvestris*  *Pyrola media*  *Pyrola rotundiflora*  *Silene otites* | 254 | 9 | 17 | 40 | 82 | 43 |
| 9410 | Acidophilous Picea forests of the montane to alpine levels (Vaccinio-Piceetea) | 43 | *Abies alba*  *Betula pendula*  *Betula pubescens ssp. pubescens*  *Picea abies*  *Pinus sylvestris*  *Populus tremola*  *Sorbus aucuparia*  *Larix decidua* | 770 | 16 | 45 | 113 | 236 | 131 |
| 9420 | Alpine Larix decidua and/or Pinus cembra forests | 43 | *Calamagrostis villosa*  *Erica carnea*  *Homogyne alpina*  *Larix decidua*  *Picea abies*  *Pinus cembra*  *Sorbus aucuparia*  *Vaccinium myrtillus*  *Vaccinium vitis- idea*  *Valeriana tripteris* | 56 | 5 | 10 | 22 | 33 | 10 |
